# Meta-analysis of prostate cancer gene expression data identifies a novel discriminatory signature enriched for glycosylating enzymes

**DOI:** 10.1186/s12920-014-0074-9

**Published:** 2014-12-31

**Authors:** Stefan J Barfeld, Philip East, Verena Zuber, Ian G Mills

**Affiliations:** Bioinformatics & Biostatistics, Cancer Research UK London Research Institute, 44 Lincoln’s Inn Fields, London, WC2A 3PX UK; Prostate Cancer Research Group, Centre for Molecular Medicine Norway (NCMM), Nordic EMBL Partnership University of Oslo and Oslo University Hospital, Oslo, Norway; Department of Cancer Prevention and Urology, Institute of Cancer Research and Oslo University Hospital, Oslo, Norway

**Keywords:** Transcription, Prostate cancer, Signature

## Abstract

**Background:**

Tumorigenesis is characterised by changes in transcriptional control. Extensive transcript expression data have been acquired over the last decade and used to classify prostate cancers. Prostate cancer is, however, a heterogeneous multifocal cancer and this poses challenges in identifying robust transcript biomarkers.

**Methods:**

In this study, we have undertaken a meta-analysis of publicly available transcriptomic data spanning datasets and technologies from the last decade and encompassing laser capture microdissected and macrodissected sample sets.

**Results:**

We identified a 33 gene signature that can discriminate between benign tissue controls and localised prostate cancers irrespective of detection platform or dissection status. These genes were significantly overexpressed in localised prostate cancer versus benign tissue in at least three datasets within the Oncomine Compendium of Expression Array Data. In addition, they were also overexpressed in a recent exon-array dataset as well a prostate cancer RNA-seq dataset generated as part of the The Cancer Genomics Atlas (TCGA) initiative. Biologically, glycosylation was the single enriched process associated with this 33 gene signature, encompassing four glycosylating enzymes. We went on to evaluate the performance of this signature against three individual markers of prostate cancer, v-ets avian erythroblastosis virus E26 oncogene homolog (ERG) expression, prostate specific antigen (PSA) expression and androgen receptor (AR) expression in an additional independent dataset. Our signature had greater discriminatory power than these markers both for localised cancer and metastatic disease relative to benign tissue, or in the case of metastasis, also localised prostate cancer.

**Conclusion:**

In conclusion, robust transcript biomarkers are present within datasets assembled over many years and cohorts and our study provides both examples and a strategy for refining and comparing datasets to obtain additional markers as more data are generated.

**Electronic supplementary material:**

The online version of this article (doi:10.1186/s12920-014-0074-9) contains supplementary material, which is available to authorized users.

## Background

Alterations in transcriptional programmes are often involved in neoplastic transformation and progression and defining these changes will help to understand the underlying biology of the malignancies. Gene Expression Microarray Analysis and more recently high-throughput RNA sequencing (RNA-seq) are commonly used techniques when trying to acquire an unbiased view of the expression levels of large numbers of genes. In order to define more compact and manageable expression modules that might predict risk or prognosis, various approaches have been used across several studies. These include the identification of consensus profiles across multiple datasets [1] and identifying biologically categorised gene sets with enriched representation of deregulated genes [2,3]. Furthermore, smaller expression modules have also been identified using hierarchical clustering methods to generate clusters containing genes with similar expression profiles across glioblastoma samples [3]. The high degree of prostate tissue heterogeneity, however, represents a challenge for transcriptomics since the relative prevalence of each cell type within a given sample determines the overall expression profile. This makes it difficult to compare prostate samples that have very different epithelial and stromal contents. Many studies have compared tumor tissue with benign hyperplastic tissue, or with non-tumoral prostate tissues that were not precisely characterised in terms of location or epithelial representation. Therefore, the outcomes of these analyses were possibly biased because the comparisons included tissues of diverse histological or embryological origins. Various approaches have been used to overcome this issue including *in silico* corrections to compensate for variable epithelial representations in different samples [4], and laser microdissection combined with *in vitro* linear amplification [5]. The laser capture microdissection study of Tomlins *et al.* yielded several informative molecular concepts (multi-gene modules), which provide a rich source of data for further refinement and follow-up as well as distinguishing between stromal and epithelial cancer signatures [5]. It is, however, not clear how detectable those concepts might be in material extracted from heterogeneous whole tissue sections, an important point given the time and expense associated with laser capture microdissection.

In this study, we have therefore set out with a number of goals. First and foremost amongst these was to determine whether we could identify gene signatures that were statistically significant in datasets generated from both whole tissue sections and laser capture microdissected material. If so, this might indicate that with the right filtering approach, sample heterogeneity might not be a completely confounding challenge to transcriptomic analysis. Secondly, if we were able to identify such signatures, we then wanted to be able to refine them to a point that the signature and any pathway or process enriched within it could be easily validated by other experimental and clinical research groups. Here, we report a concise 33-gene signature with biological enrichment for glycosylation, which discriminates between benign tissue and prostate cancer (PCa) across multiple transcript detection platforms and sample types.

## Methods

### Description of datasets

Five datasets were downloaded and used in this study.

1. A 19-sample dataset generated by Varambally *et al.,* using the Affymetrix Human Genome U133 Plus 2.0 Array platform. The dataset consisted of 13 macrodissected individual benign prostate, primary and metastatic PCa samples and 6 pooled samples from benign, primary or metastatic PCa tissues. The expression array data were downloaded from GEO under accession number GSE3325 (http://www.ncbi.nlm.nih.gov/geo/query/acc.cgi?acc=GSE3325).
2. A 104-sample dataset generated by Tomlins *et al.,* using an in-house cDNA microarray platform (Chinnaiyan Human 20K Hs6). Laser capture microdissection was used to isolate 101 specific cell populations from 44 individuals representing PCa progression in a range of sample categories encompassing 12 stromal and 89 epithelial cell populations. These were subcategorised as EPI_BPH (benign prostatic hyperplasia epithelium), EPI_ADJ_PCA (normal epithelium adjacent to PCa), EPI_ATR (atrophic epithelium – simple atrophy), EPI_ATR_PIA (atrophic epithelium), PIN (prostatic intraepithelial neoplasia), PCA (prostate carcinoma), MET_HN (Metastatic Prostate Carcinoma - Hormone Naïve), MET_HR (Metastatic Prostate Carcinoma - Hormone Refractory), STROMA_EPIBPH (BPH Stroma - Epithelial BPH ), STROMA_NOR (Normal Stroma - Organ Donor), STROMA_ADJ_PCA (Normal Stroma - Adjacent to prostate cancer). In addition three samples were EPI_NOR (Normal Epithelium - Organ Donor). In our study we maintain this nomenclature in describing the dataset. The expression array data were downloaded from GEO under accession number GSE6099 (http://www.ncbi.nlm.nih.gov/geo/query/acc.cgi?acc=GSE6099).
3. A multi-cancer microarray dataset generated by Ramaswamy *et al.,* and consisting of 218 tumour samples, spanning 14 common tumour types, and 90 normal tissue samples and profiled on Affymetrix oligonucleotide microarrays (Hu6800 and Hu35KsubA GeneChips). The 14 tumour types incorporated into this study were breast adenocarcinoma, prostate adenocarcinoma, lung adenocarcinoma, colorectal adenocarcinoma, lymphoma, bladder transitional cell carcinoma, melanoma, uterine adenocarcinoma, leukemia, renal cell carcinoma, pancreatic adenocarcinoma, ovarian adenocarcinoma, pleural mesothelioma and cancers of the central nervous system. The dataset was downloaded from the Broad Institute website (http://www.broadinstitute.org/cgi-bin/cancer/publications/pub_paper.cgi?mode=view&paper_id=61).
4. A PCa dataset generated by Taylor *et al.,* for 150 tumours, 29 matched normal samples, and 6 cell lines using the Affymetrix Human Exon 1.0 ST array platform. There were 27 metastatic samples amongst the 150 tumours and 35 cases of biochemical relapse (Additional file 1 in Taylor *et al.,*). The expression array data were downloaded from GEO under accession number GSE21034 (http://www.ncbi.nlm.nih.gov/geo/query/acc.cgi?acc=GSE21034).
5. High throughput RNA sequencing data were generated by the The Cancer Genomics Atlas (TCGA) consortium for 383 samples, 50 benign samples and 333 primary tumours. 48 of these samples represented advanced disease with Gleason grade ≥8 and 13 cases had undergone progression as characterised by post-operative biochemical recurrence. Data were downloaded from the UCSC Cancer Genome Browser (https://genome-cancer.ucsc.edu/) - TCGA_PRAD_exp_HiSeqV2-2014-05-02.tgz. Associated clinical data were downloaded from the TCGA Data Portal (https://tcga-data.nci.nih.gov/tcga/).
6. A PCa dataset generated by Grasso *et al.,* generated for 28 benign prostate tissue samples, 59 localised PCa and 35 metastatic PCa was generated on two Agilent microarray platforms (whole genome microarray (4x44K, G4112F) and whole human genome oligo microarray (G4112A ). The expression array data were downloaded from GEO under accession number GSE35988 (http://www.ncbi.nlm.nih.gov/geo/query/acc.cgi?acc=GSE35988).
7. Additional datasets were interrogated for the expression of individual genes within signatures through the Oncomine Compendium of Expression Array data (www.oncomine.org).

### Prostate cancer dependent expression changes

To generate an initial broad progression dependent gene set, we used the prostate progression expression dataset GSE3325 (NCBI GEO database). We quantile normalised probe level intensity values and generated probe set signal estimates using RMA (1,2). We first characterised reporters with a coefficient of variance of less than or equal to 0.05 as uninformative and removed them from further analysis. Reporters having intensities below the 10^th^ quantile (3.91) in more than 75% of the samples were also removed. We identified progression associated expression changes by linear model. Primary tumour versus benign and metastatic versus primary contrasts were run and differential reporters identified using a 0.01 FDR threshold. Reporters were further filtered selecting those with a differential effect size of greater than or equal to 2-fold. This resulted in a progression signature set of 4662 reporters, 3021 genes (121 primary, 2900 metastatic, primary ∩ metastatic = 102). Signatures derived from this primary dataset were subsequently applied to two additional datasets, one prostate dataset generated by Tomlins *et al*. [5] in a laser capture microdissection study (GSE6099) and another generated by Ramaswamy *et al.* [6] and representing multi-tissue primary tumours and metastases (accessible through the Broad Institute data repository: http://www.broadinstitute.org/cgi-bin/cancer/datasets.cgi).

### Identifying correlated gene modules

We clustered our progression gene set using hierarchical clustering with a Ward agglomerative method designed to minimize intra-cluster variance (hclust, Bioconductor) and a 1 - Pearson correlation coefficient dissimilarity measure. We found this method produced a more highly correlated clustering structure when compared to other methods leading to more compact sub-clusters (Additional file 1). We characterised correlated gene modules by cutting the cluster dendrogram at branch lengths ranging from log10(0.05) to log10(3000) giving 39 equal intervals across the log scale. We removed clusters containing less than 3 members from further analysis. We selected modules defined at branch lengths 0f 0.6, 0.8, 1.1, 1.9, 2.5, 4.5, 10.6, 24.7 and 101.6 for further analysis since these gave a broad range of cluster numbers. Since a smaller branch length threshold does not always sub divide a parent module modules can be duplicated at different thresholds. These were removed from further analysis assigning them to the largest branch threshold at which they appears. We assigned Gene Ontology classifications to modules by testing for enrichment at GO nodes using a hyper-geometric distribution and a 0.01 p-value threshold. We carried out this analysis at the gene level by translating chip reporter probeset ids to Entrez gene ids. All reporters from the progression signature with assigned Entrez gene ids were used as background. Analysis was carried out using the GoStats package, Bioconductor.

### Phenotype dependent transcript module expression changes

To determine differential regulation of modules within other expression datasets we first identified phenotype dependent expression changes for each sample using an absolute fold change filter of greater than 2. To generate fold changes against which we could filter each gene was scaled to a baseline intensity value. In the case of dataset GSE3325 each signal intensity from the primary tumour samples was scaled to the corresponding median gene signal intensity across the benign tumour samples. Likewise all metastatic samples were scaled to the median across all primary tumour samples. Prior to mapping modules to dataset GSE6099 we background corrected each sample using a normexp method and print tip loess normalised (normalizeWithinArrays(), Bioconductor). We then scaled PIN samples to EPI_ADJ_PCA control samples, PCA samples to PIN, MET_HNF to PCA and MET_HR to PCA. To identify hormone refractory dependent expression changes MET_HR samples were scaled to the median across the non-refractory samples MET_HN. To determine module induction or repression within the scaled samples we tested for enrichment of module genes within the sample associated expression changes using a hypergeometric distribution, <= 0.05 fdr. Mapping was achieved across array platforms using NCBI Entrez gene ids. Modules with an intersection of less than 3 were discarded from the analysis.

### Phenotype segregation

To determine if any of the enriched modules were capable of segregating samples on phenotype we built contingency tables across clinical conditions from each of the data sets (Tomlins [5] and Ramaswamy [6]) for induced and repressed modules and tested for sample enrichment using a Fisher’s Exact test. Here we tested each phenotypic group against all others from each data set.

### Cluster analysis

To determine the best clustering method and branch length thresholds to apply to our analysis we clustered our 4662 reporter prostate tumour progression signature (Additional file 2: Table S1) using single, average, complete, Ward’s^1^ minimum variance method and mcquitty^2^ agglomerative hierarchical clustering methods along with a divisive method. The hclust function from Bioconductor^3^ was used for the agglomerative techniques and the diana function from the cluster package from Bioconductor was used to run the divisive method. We used the cutree function at branch length thresholds ranging from 0.05 to 2 in increments of 0.05 to derive groups of correlated genes. In the case of the Ward agglomerative method where the branch scales [ it is unclear how Ward calculates its branch lengths, need to find out ] branch length thresholds ranged from log_10_(0.05) to log_10_(3000) in increments of log_10_(3000/0.05)/39. Branch length threshold intervals were chosen to produce a broad range of cluster numbers.

### Cluster correlation

To assess the extent to which genes assigned to clusters are correlated we calculated a within-cluster dissimilarity value for each cluster^4^. This is given by

$$ W\ C=\frac{1}{K}{\displaystyle \sum_{k=1}^K\frac{1}{2{\mathrm{N}}_k}}{\displaystyle \sum_{i=1}^N{\displaystyle \sum_{j=1}^Nd\ {x}_i,{x}_j}} $$

where *d x*_*i*_, *x*_*j*_ is the dissimilarity between genes _*i*_ and _*j*_ across all samples, *i,j =* 1,2,*…N* where *N* is the total number of cluster members and *k =* 1,2,*…,K* where *K* is the total number of clusters. In our case the dissimilarity measure is 1 – Pearson correlation coefficient between 2 genes across all samples. *W(C)* dissimilarity values across the array of branch length thresholds can be seen plotted against cluster number in Additional file 1: Figure S1. As observed the Ward agglomerative method out performs all other methods producing clusters that are less dissimilar and therefore more highly correlated than those generated from other methods relative to the number of clusters produced. These results provide a justification for using hierarchical clustering with a ward agglomerative method to generate sets of co-regulated genes.

### Cluster gene ontology entropy

To quantify the information content of our clusters from a biological perceptive we assigned GO terms to cluster members. This was achieved by mapping GO terms via reporter entrez gene id assignments using the GO.db annotation package from Bioconductor. To quantify the GO information content of a cluster we calculated Shannon Entropy bit values given by:

$$ H\ X=-\frac{1}{K}{\displaystyle \sum_{k=1}^K{\displaystyle \sum_{i=1}^Np\ {x}_i\ { \log}_bp{x}_i}} $$

where *x*_*i*_ is a cluster associated GO term, p(*x*_*i*_*)* is the probability of choosing *x*_*i*_ from all cluster GO terms , *i = 1,2,…N* where *N* is the total number of unique cluster GO terms, *k = 1,2,…K* where K is the total number of clusters and *b = 2. H(X)* bit values for different branch length thresholds can be seen plotted against cluster number for the different clustering techniques in Additional file 3: Figure S2. As observed the Ward clustering method produces clusters with higher GO bit values when compared to other methods. This implies greater uncertainly in the GO term mappings for clusters generated by the ward method thus indicating the production of clusters more GO information rich when compared to other methods. This provides further justification for using hierarchical clustering with a ward agglomerative method to generate sets of co-regulated genes.

### Visualization of gene signatures through heatmaps

For visualization, sample groups were averaged using the mean prior to high level mean and variance normalization using the freely available software J-Express 2012 (http://jexpress.bioinfo.no/site/). Subsequently, both sample groups and genes were hierarchically clustered using complete linkage and Euclidian distance using the freely available software Cluster 3.0 (http://bonsai.hgc.jp/~mdehoon/software/cluster/software.htm). Heatmaps were produced using Java Tree View (http://jtreeview.sourceforge.net/).

### Evaluating gene signature specificity and sensitivity

Testset: Grasso 1 Platform GPL6480

- # Title Agilent-014850 Whole Human Genome Microarray 4x44K G4112F
- # tissue: benign prostate tissue (N)
- # 12
- # tissue: localised prostate cancer (T)
- # 49
- # tissue: metastatic castrate resistant prostate cancer (WA)
- # 27.

Training data: Grasso 2 Platform GPL6848

- # Title Agilent-012391 Whole Human Genome Oligo Microarray G4112A
- # tissue: benign prostate tissue (N)
- # 16
- # tissue: localised prostate cancer (T)
- # 10
- # tissue: metastatic castrate resistant prostate cancer (WA)
- # 8.

To evaluate the prediction performance 33 gene signature we analysed data from a microarray experiment of Grasso et al as available from GEO (GSE35988) http://www.ncbi.nlm.nih.gov/geo/query/acc.cgi?acc=GSE35988.

The dataset includes measurements on two different microarray platforms GPL6480 and GPL6848 and includes three different tissue types (benign, localised and metastatic castrate resistant prostate cancer).

We used the samples typed on platform GPL6848 as trainings data to derive the weights of the genes in the multi-gene signature. First, we replaced missing values using the k nearest neighbor algorithm as implemented in the R package impute. The gene PCA3 was not measured on the microarray and the gene CRISP3 was not observed in more than half of these samples. Thus, these two genes were excluded from the signature. We estimated the weights of each gene using an L2 regularized logit regression model [7] with the R package glmnet.

Then we used the samples typed on platform GPL6480 as test data to evaluate the prediction performance of the gene-signature. Per sample we computed one score as the weighted average over the 31 proposed genes where the weights were defined by the independent training data. Finally, we computed ROC statistics and report the area under the curve (AUC) of the ROC curve (R package ROCR). The AUC indicates the ability of a marker to distinguish between two groups, where a value 0.5 is random and a value of 1 represents a perfect distinction between the groups. Additionally, we were looking at the AUC of specific genes, in particular KLK3, ERG and AR.

## Results and discussion

In order to define our starting signatures, we selected a dataset published by Varambally *et al*. about a decade ago and consisting of a small number of whole tissue sections [8]. This constitutes the smallest and oldest dataset used in our meta-analysis. It was, however, extensively validated at both the transcript and protein level in the original study and therefore provides a high-degree of confidence in data quality. We chose to define our starting signatures using this dataset in order to assess how much information could be derived despite the limitations in size and age. Within these data, we firstly identified transcripts that were differentially expressed in localised prostate versus benign tissue or in metastatic disease versus localised cancer using a conventional linear model approach. This approach identified 121 genes differentially expressed in localised primary cancers (primary versus benign, 0.01 FDR) and 2900 genes associated with metastatic status (metastatic versus primary, 0.01 FDR), which were covered by 4662 probes in total (Additional file 2: Table S1). To further refine these gene lists into discrete signatures, we constructed a gene coexpression network using Pearson correlation coefficients and hierarchical clustering using the Ward agglomerative method (**See****Methods****section**).

A number of different correlation or dissimilarity metrics have been employed when constructing co-expression networks. To determine the correlation between the genes we used a Pearson correlation coefficient to construct a dissimilarity matrix across all affected samples in the prostate tumour progression dataset and all genes identified in the preliminary analysis. We then used hierarchical clustering to group the genes. There are a number of available agglomeration methods available each producing their own clustering structure. To determine the best agglomeration method to apply in constructing our expression modules, we clustered our prostate tumour progression signature using single, average, complete, the Ward [9] minimum variance method and the Mcquitty [10] agglomerative hierarchical clustering method along with a divisive method. The performance of these clustering methods by using an algorithm to determine the extent to which genes assigned to clusters are correlated generating a within-cluster dissimilarity value for each cluster (**Methods****section – ‘****Cluster Correlation****’**). In addition, we assessed the information content in gene ontology terms associated with clusters generated using each method by calculating Shannon Entropy bit values (**Methods****section** - ‘**Cluster Gene Ontology Entropy****’)**. Shannon entropy and coefficient of variation are well known in a great many application domains, from theoretical physics to computational chemistry to materials science. They have been applied in bioinformatics as well, most notably in statistical genetics and molecular biology. Shannon entropy is derived from information theory [11]. Most relevant for this study the approach has previously been used as a measure of the robustness of gene regulatory networks [12], to accelerate feature elimination when classifying microarray expression data [13]. By these measures the Ward clustering method provided both more tightly associated coexpressed gene clusters as well as clusters with higher GO bit values when compared to other methods, indicative of greater information content in the ontologies derived for coexpression clusters generated using the Ward approach than using the other approaches. Additional file 4: Table S2 provides a complete list of coexpressed genes signatures generated used the Ward approach at all branching thresholds.

Four large gene signatures were generated at the least stringent cut-point consisting of 1334 genes referred to as signature 1 (annotated as 101.6.1: Additional file 5: Table S3), 652 genes referred to as signature 2 (annotated as 101.6.2: Additional file 6: Table S4), 836 genes referred to as signature 3 (annotated as 101.6.3: Additional file 7: Table S5) and 357 genes referred to as signature 4 (annotated as 101.6.4: Additional file 8: Table S6). Signatures 1 and 2 contained genes that predominantly discriminated between localised PCa and benign tissue. Using DAVID ontology enrichment search (http://david.abcc.ncifcrf.gov/) to determine whether KEGG pathways were enriched within these signatures, we identified focal adhesions (hsa04510: Focal adhesion, p-value 7.21x10^−6^) (Table 1) as the most significant pathway for signature 1and complement and coagulation cascades for signature 2 (hsa04610: Complement and coagulation cascades, p-value 0.002) (Table 2). The 38 genes associated with the focal adhesion annotation (p-value 7.21x10^−6^) in signature 1 are listed in Table 1 and were all significantly downregulated in metastatic samples relative to benign and localised PCa. Half of these genes were laminins (eg. laminin alpha subunit-4 (LAMA4)), integrins (eg. integrin, alpha 1 (ITGA1) and five others), thrombospondins (thrombospondins 1 and 4 (THBS1/4), collagens, actins and myosins which may reflect the remodelling of the extracellular matrix and loss of stroma in particular during the transition to metastasis. The enrichment for complement and coagulation cascades in signature 2 (p value 0.002) included complement (eg. C1R, C1QA, C3) and plasma factors as well as serpin peptidase inhibitor as listed in Table 2 and were also predominantly downregulated in metastatic cases versus benign tissue and localised PCa. Collectively, these pathway enrichments might reflect a combination of extracellular matrix changes and the contribution of infiltrating immune cells and the inflammatory response. However, given that the Varambally dataset consists of whole-tissue sections it is not possible in this meta-analysis to precisely attribute these signatures to a particular biological process.

**Table 1:** **KEGG pathway enrichment analysis for the genes comprising signature 1 (101.6.1)**

**Category**	**Term**	**Count**	**%**	**PValue**	**Genes**	**List Total**	**Pop Hits**	**Pop Total**	**Fold Enrichment**	**Bonferroni**	**Benjamini**	**FDR**
KEGG_PATHWAY	hsa04510:Focal adhesion	37	2.86	2.01E-06	CAV2, CAV1, MYL5, MYL2, TNC, PTEN, MYL9, VCL, IGF1R, LAMB3, LAMB2, ITGB8, ILK, ITGB6, PDGFC, PAK1, THBS1, THBS4, COL4A4, PRKCA, ACTB, MET, ITGA1, ACTN1, IGF1, HGF, COL4A6, FLNA, LAMA4, ITGA6, CCND2, ITGA5, JUN, ITGA8, COL1A2, MYLK, PTENP1, PARVA	404	201	5085	2.32	3.42E-04	3.42E-04	0
KEGG_PATHWAY	hsa05414:Dilated cardiomyopathy	21	1.62	2.37E-05	ACTB, SLC8A1, ACTC1, MYL2, LMNA, ITGA1, IGF1, CACNB2, TPM2, TPM1, TPM4, TGFB2, DES, ITGA6, ITGA5, ITGB8, PLN, ITGA8, ITGB6, PRKACB, SGCB	404	92	5085	2.87	0	0	0.03
KEGG_PATHWAY	hsa05410:Hypertrophic cardiomyopathy (HCM)	20	1.55	2.50E-05	ACTB, SLC8A1, ACTC1, IL6, MYL2, LMNA, ITGA1, IGF1, CACNB2, TPM2, TPM1, TPM4, TGFB2, DES, ITGA6, ITGA5, ITGB8, ITGA8, ITGB6, SGCB	404	85	5085	2.96	0	0	0.03
KEGG_PATHWAY	hsa00280:Valine, leucine and isoleucine degradation	12	0.93	4.71E-04	MCCC2, ALDH7A1, ALDH1B1, MCEE, AOX1, BCKDHB, DLD, ACAD8, ACAT1, HIBADH, ALDH3A2, AUH	404	44	5085	3.43	0.08	0.02	0.57
KEGG_PATHWAY	hsa04512:ECM-receptor interaction	17	1.31	7.54E-04	COL4A4, TNC, ITGA1, COL4A6, CD47, LAMA4, LAMB3, LAMB2, CD44, ITGA6, ITGB8, ITGA5, ITGA8, ITGB6, COL1A2, THBS1, THBS4	404	84	5085	2.55	0.12	0.03	0.92
KEGG_PATHWAY	hsa04270:Vascular smooth muscle contraction	20	1.55	0	PRKCA, ACTA2, PPP1R12B, CALD1, MRVI1, KCNMB1, ITPR1, MYL9, ITPR2, EDNRA, AGTR1, ACTG2, PLCB4, GNAQ, PLA2G12A, MYH11, PRKACB, PLCB1, PPP1R14A, MYLK	404	112	5085	2.25	0.17	0.03	1.35
KEGG_PATHWAY	hsa05412:Arrhythmogenic right ventricular cardiomyopathy (ARVC)	14	1.08	0.01	ACTB, SLC8A1, LMNA, ITGA1, ACTN1, CACNB2, DES, ITGA6, ITGB8, ITGA5, PKP2, ITGA8, ITGB6, SGCB	404	76	5085	2.32	0.65	0.14	7.2
KEGG_PATHWAY	hsa04610:Complement and coagulation cascades	13	1.01	0.01	C4A, MASP1, C4B, CFB, C1S, CD59, KLKB1, F3, SERPINE1, SERPINA1, CFI, C2, PROS1	404	69	5085	2.37	0.71	0.14	8.49
KEGG_PATHWAY	hsa04310:Wnt signaling pathway	22	1.7	0.01	PRKCA, CSNK1A1, WNT5B, CAMK2G, MMP7, FZD1, DKK1, PLCB4, SFRP1, CCND2, SFRP2, JUN, SFRP4, PRICKLE2, PPP3CB, CAMK2D, WIF1, PRKACB, AXIN2, PLCB1, MYC, APC	404	151	5085	1.83	0.72	0.13	8.79
KEGG_PATHWAY	hsa05332:Graft-versus-host disease	9	0.7	0.01	HLA-DQB1, IL6, HLA-DRB1, HLA-DRB4, HLA-C, HLA-DPA1, HLA-B, FAS, HLA-DMB, HLA-G, HLA-DQA1	404	39	5085	2.9	0.82	0.16	11.58
KEGG_PATHWAY	hsa00590:Arachidonic acid metabolism	11	0.85	0.01	CYP2U1, GPX2, PTGIS, PTGS2, PTGDS, ALOX15B, PLA2G12A, PTGS1, GGTLC3, GGT1, ALOX5, CBR3	404	56	5085	2.47	0.86	0.16	13.08
KEGG_PATHWAY	hsa00480:Glutathione metabolism	10	0.77	0.02	GSTM1, GPX2, GSTM2, GSTA4, GGTLC3, GSTZ1, GSTO2, ANPEP, GSTT2, GGT1, GSTO1	404	50	5085	2.52	0.93	0.2	17.11
KEGG_PATHWAY	hsa04514:Cell adhesion molecules (CAMs)	19	1.47	0.02	HLA-DQB1, HLA-DRB1, CDH1, ITGB2, HLA-C, NEO1, HLA-B, HLA-DMB, CDH3, HLA-DQA1, HLA-G, ITGA6, ITGB8, ITGA8, PVRL3, CD2, HLA-DRB4, HLA-DPA1, JAM2, NEGR1, SELE	404	132	5085	1.81	0.93	0.19	17.38
KEGG_PATHWAY	hsa04940:Type I diabetes mellitus	9	0.7	0.02	HLA-DQB1, HLA-DRB1, PTPRN2, HLA-DRB4, HLA-C, HLA-DPA1, HLA-B, FAS, HLA-DMB, HLA-G, HLA-DQA1	404	42	5085	2.7	0.93	0.17	17.49
KEGG_PATHWAY	hsa04350:TGF-beta signaling pathway	14	1.08	0.02	SMAD6, FST, DCN, TGFB2, ACVR2A, ID1, ZFYVE16, ID4, ID3, THBS1, MYC, BMPR1A, ACVR1, THBS4	404	87	5085	2.03	0.96	0.19	20.39
KEGG_PATHWAY	hsa04810:Regulation of actin cytoskeleton	27	2.09	0.02	FGFR2, FGF7, MYL5, MYL2, DIAPH2, FGF13, ITGB2, MYL9, VCL, GSN, ITGB8, ITGB6, RRAS, PDGFC, PAK1, FGF2, APC, ACTB, LIMK2, ITGA1, ACTN1, ITGA6, CHRM3, ITGA5, CFL2, ITGA8, MYLK	404	215	5085	1.58	0.96	0.18	20.54
KEGG_PATHWAY	hsa05330:Allograft rejection	8	0.62	0.02	HLA-DQB1, HLA-DRB1, HLA-DRB4, HLA-C, HLA-DPA1, HLA-B, FAS, HLA-DMB, HLA-G, HLA-DQA1	404	36	5085	2.8	0.97	0.19	22.63
KEGG_PATHWAY	hsa00330:Arginine and proline metabolism	10	0.77	0.02	ALDH7A1, ALDH18A1, ACY1, GATM, GLUD2, ALDH1B1, MAOB, OAT, ALDH3A2, CKB	404	53	5085	2.37	0.98	0.19	23.67
KEGG_PATHWAY	hsa00982:Drug metabolism	11	0.85	0.02	GSTM1, GSTM2, CYP3A5, GSTA4, AOX1, MAOB, ADH5, GSTZ1, GSTO2, GSTT2, GSTO1	404	62	5085	2.23	0.98	0.18	24.32
KEGG_PATHWAY	hsa05218:Melanoma	12	0.93	0.02	FGF7, MET, IGF1, CDH1, CDK6, FGF13, HGF, RB1, PTEN, IGF1R, PDGFC, FGF2, PTENP1	404	71	5085	2.13	0.98	0.18	24.47
KEGG_PATHWAY	hsa05416:Viral myocarditis	12	0.93	0.02	ACTB, HLA-DQB1, CAV1, HLA-DRB1, ITGB2, HLA-C, HLA-B, HLA-DMB, HLA-DQA1, HLA-G, MYH11, HLA-DRB4, HLA-DPA1, SGCB	404	71	5085	2.13	0.98	0.18	24.47
KEGG_PATHWAY	hsa05310:Asthma	7	0.54	0.02	FCER1A, HLA-DQB1, HLA-DRB1, HLA-DRB4, FCER1G, HLA-DPA1, HLA-DMB, HLA-DQA1	404	29	5085	3.04	0.98	0.18	25.37
KEGG_PATHWAY	hsa00640:Propanoate metabolism	7	0.54	0.04	ALDH7A1, ALDH1B1, MCEE, SUCLA2, ACAT1, ACSS3, ALDH3A2	404	32	5085	2.75	1	0.25	36.89
KEGG_PATHWAY	hsa04916:Melanogenesis	14	1.08	0.05	PRKCA, WNT5B, GNAI1, CAMK2G, CREB1, EDN1, FZD1, EDNRB, PLCB4, GNAQ, CAMK2D, CREB3L4, PRKACB, PLCB1	404	99	5085	1.78	1	0.3	44.76
KEGG_PATHWAY	hsa04020:Calcium signaling pathway	21	1.62	0.06	PRKCA, SLC8A1, CAMK2G, PHKA1, PTGFR, ITPR1, ITPR2, EDNRA, AGTR1, EDNRB, GNAL, CD38, PLCB4, GNAQ, CHRM3, PLN, CAMK2D, PPP3CB, PRKACB, PLCB1, MYLK	404	176	5085	1.5	1	0.37	54.64
KEGG_PATHWAY	hsa04530:Tight junction	17	1.31	0.06	PRKCA, ACTB, RAB3B, MYL5, MAGI2, ZAK, MYL2, MPDZ, GNAI1, ACTN1, AMOTL1, PTEN, MYL9, EPB41L2, MYH11, RRAS, JAM2, PTENP1	404	134	5085	1.6	1	0.36	54.73
KEGG_PATHWAY	hsa05222:Small cell lung cancer	12	0.93	0.07	COL4A4, PTGS2, CDK6, RB1, PTEN, COL4A6, LAMB3, LAMA4, LAMB2, ITGA6, PIAS1, MYC, PTENP1	404	84	5085	1.8	1	0.36	56.55
KEGG_PATHWAY	hsa04360:Axon guidance	16	1.24	0.08	LIMK2, GNAI1, MET, NTN4, SLIT2, EPHA3, SEMA5A, EPHA4, EPHB6, CFL2, PPP3CB, SEMA3C, EFNA5, UNC5D, PAK1, RASA1	404	129	5085	1.56	1	0.42	65.75
KEGG_PATHWAY	hsa04115:p53 signaling pathway	10	0.77	0.09	SERPINB5, CCND2, SERPINE1, IGF1, CDK6, FAS, GADD45B, THBS1, CCNG2, PTEN, PTENP1	404	68	5085	1.85	1	0.42	66.77
KEGG_PATHWAY	hsa04720:Long-term potentiation	10	0.77	0.09	PRKCA, PLCB4, GNAQ, CAMK2G, CAMK2D, PPP3CB, PRKACB, PLCB1, ITPR1, ITPR2	404	68	5085	1.85	1	0.42	66.77
KEGG_PATHWAY	hsa04672:Intestinal immune network for IgA production	8	0.62	0.09	HLA-DQB1, IL6, TNFSF13B, HLA-DRB1, HLA-DRB4, HLA-DPA1, HLA-DMB, HLA-DQA1, TGFB2	404	49	5085	2.05	1	0.42	68.02
KEGG_PATHWAY	hsa05322:Systemic lupus erythematosus	13	1.01	0.09	HLA-DQB1, HLA-DRB1, C4A, C4B, ACTN1, SSB, C1S, H2AFJ, HLA-DMB, HLA-DQA1, HLA-DRB4, HLA-DPA1, H3F3B, C2	404	99	5085	1.65	1	0.41	68.42
KEGG_PATHWAY	hsa00620:Pyruvate metabolism	7	0.54	0.09	ALDH7A1, ALDH1B1, DLD, ACYP2, DLAT, ACAT1, ALDH3A2	404	40	5085	2.2	1	0.41	69.45
KEGG_PATHWAY	hsa04730:Long-term depression	10	0.77	0.09	PRKCA, IGF1R, PLCB4, GNAQ, GNAI1, PLA2G12A, IGF1, PLCB1, ITPR1, ITPR2	404	69	5085	1.82	1	0.4	69.5
KEGG_PATHWAY	hsa00980:Metabolism of xenobiotics by cytochrome P450	9	0.7	0.1	GSTM1, GSTM2, CYP3A5, GSTA4, ADH5, GSTZ1, GSTO2, GSTT2, GSTO1	404	60	5085	1.89	1	0.42	71.98

Genes comprising signature 1 (Additional file 5: Table S3) were uploaded into the DAVID gene ontology search engine (http://david.abcc.ncifcrf.gov/). KEGG pathway enrichment was generated and the table represents the output file ranked based on significance and annotated by column header.

**Table 2:** **KEGG pathway enrichment analysis for the genes comprising signature 2 (101.6.2)**

**Category**	**Term**	**Count**	**%**	**PValue**	**Genes**	**List Total**	**Pop Hits**	**Pop Total**	**Fold Enrichment**	**Bonferroni**	**Benjamini**	**FDR**
KEGG_PATHWAY	hsa04610:Complement and coagulation cascades	10	1.56	0	C1QA, FGG, A2M, C3, KLKB1, CD46, C1R, SERPING1, C1S, CFD	219	69	5085	3.37	0.33	0.33	2.97
KEGG_PATHWAY	hsa04540:Gap junction	10	1.56	0.01	TJP1, ADCY2, GNAI1, PDGFA, TUBB6, GUCY1A3, GJA1, LPAR1, PRKACB, ITPR2	219	89	5085	2.61	0.88	0.65	14.98
KEGG_PATHWAY	hsa04142:Lysosome	11	1.72	0.03	AGA, HGSNAT, LAMP2, CTSK, GM2A, PSAP, LGMN, CTSB, SCARB2, FUCA1, CLN5	219	117	5085	2.18	0.99	0.77	28.69
KEGG_PATHWAY	hsa04270:Vascular smooth muscle contraction	10	1.56	0.05	PLA2G4A, ADCY2, CALD1, MRVI1, GUCY1A3, PRKCH, PRKACB, PPP1CB, MYLK, ITPR2	219	112	5085	2.07	1	0.87	46.04
KEGG_PATHWAY	hsa04310:Wnt signaling pathway	12	1.88	0.06	CCND1, PRICKLE1, CCND2, BTRC, NFAT5, CAMK2D, TP53, MAPK10, PRKACB, FZD5, FZD4, FZD7	219	151	5085	1.85	1	0.85	51.51
KEGG_PATHWAY	hsa05330:Allograft rejection	5	0.78	0.07	HLA-DRB5, HLA-DPB1, HLA-E, HLA-DOA, HLA-DRA	219	36	5085	3.22	1	0.84	56.25
KEGG_PATHWAY	hsa05416:Viral myocarditis	7	1.1	0.08	CAV1, CCND1, HLA-DRB5, HLA-DPB1, HLA-E, HLA-DOA, HLA-DRA	219	71	5085	2.29	1	0.86	64.57
KEGG_PATHWAY	hsa05332:Graft-versus-host disease	5	0.78	0.08	HLA-DRB5, HLA-DPB1, HLA-E, HLA-DOA, HLA-DRA	219	39	5085	2.98	1	0.82	65.24
KEGG_PATHWAY	hsa04510:Focal adhesion	14	2.19	0.09	CAV1, PDGFA, MAPK10, FLNC, PPP1CB, VCL, CCND1, CCND2, ITGAV, COL6A2, RAP1A, THBS1, PIK3R1, MYLK	219	201	5085	1.62	1	0.81	67.5

Genes comprising signature 2 (Additional file 6: Table S4) were uploaded into the DAVID gene ontology search engine (http://david.abcc.ncifcrf.gov/). KEGG pathway enrichment was generated and the table represents the output file ranked based on significance and annotated by column header.

By contrast, signatures 3 and 4 contained genes that predominantly discriminated between metastatic cases and benign tissue samples. The dominant pathway for signature 3 was cell cycle regulation (hsa04110: Cell cycle, p-value 9x10^−20^) and the enrichment arose from the overexpression of a total of 36 genes linked to this process in the metastatic cases versus benign tissue. The genes are listed in Table 3 and included E2F transcription factors, DNA replication licensing factors, cyclin-dependent kinase inhibitors, cell division cycle genes and components of the mitotic spindle checkpoint control apparatus. Many of these overexpressed genes also constitute a prognostic cell cycle progression gene signature, which has been validated at the transcript level in biopsy samples [14]). For signature 4, steroid biosynthesis was the most enriched pathway (hsa00100: Steroid biosynthesis, p-value 0.03 – squalene epoxidase (SQLE), farnesyl-diphosphate farnesyltransferase 1 (FDFT1), sterol-C4-methyl oxidase-like gene (SC4MOL). In this case the enrichment was due to the differential expression of three genes that are functionally tightly linked in some cases on consecutive steps in the cholesterol biosynthesis pathway. FDFT1 was overexpressed, SC4MOL was downregulated and SQLE showed a switch in expression in which one probe on the array was repressed and another was overexpressed (Table 4). Downregulation occured predominantly in localized PCa relative to benign tissue and expression seemed higher in metastatic cases than localized prostate cancers. FDFT1 overexpression, and increases in the expression of one probe for SQLE, were most significant in the metastatic cases compared to benign tissue and localised disease. These are enzymes associated with cholesterol biosynthesis in particular and collectively catalyse 3 out of 4 consecutive reactions in the conversion of farnesyl pyrophosphate to lathosterol via squalene. FDFT1 catalyses the production of squalene from farnesyl pyrophosphate, SQLE catalyses the conversion of squalene to 2,3-epoxysqualene and SC4MOL catalyses the conversion of lanosterin to lathosterol. The two metabolites consecutively further downstream in the pathway are dehydrocholesterol and cholesterol. FDFT1 overexpression has previously been associated with aggressive PCa [15]). This is particularly intriguing since metastatic PCa is characterised by increases in the proliferative index of tumours [16] and the ability to produce autocrine steroid hormones from cholesterol in order to maintain androgen receptor activity [17]. Consequently the observation of increased levels of these enzymes in metastatic cases may hypothetically imply enhanced cholesterol biosynthesis to sustain its use for steroid hormone biogenesis by the tumours.

**Table 3:** **KEGG pathway enrichment analysis for the genes comprising signature 3 (101.6.3)**

**Category**	**Term**	**Count**	**%**	**PValue**	**Genes**	**List Total**	**Pop Hits**	**Pop Total**	**Fold Enrichment**	**Bonferroni**	**Benjamini**	**FDR**
KEGG_PATHWAY	hsa04110:Cell cycle	36	0.47	9.03E-20	E2F1, E2F2, E2F3, TTK, CHEK1, PTTG1, CCNE2, CCNE1, CDKN2A, MCM7, CDKN2C, CDKN2D, ORC6L, TFDP2, BUB1, CCNA2, STAG1, CDC7, CDC6, RBL1, SKP2, ESPL1, CDC20, MCM2, CDC25C, MCM4, CDC25A, CDC25B, CDKN1C, CCNB1, CCNB2, MAD2L1, PLK1, GSK3B, BUB1B, MAD2L2	225	125	5085	6.51	1.29E-17	1.29E-17	1.07E-16
KEGG_PATHWAY	hsa03030:DNA replication	12	0.16	2.15E-07	RFC5, PRIM1, MCM7, RFC4, POLE2, LIG1, POLA1, POLA2, MCM2, RNASEH2A, MCM4, FEN1	225	36	5085	7.53	3.08E-05	1.54E-05	2.55E-04
KEGG_PATHWAY	hsa04114:Oocyte meiosis	18	0.23	4.82E-06	SGOL1, AURKA, CDC20, ESPL1, PTTG1, CDC25C, CCNE2, CCNB1, CCNE1, CCNB2, MAD2L1, ADCY9, CALML3, PLK1, BUB1, FBXO5, CAMK2B, MAD2L2	225	110	5085	3.7	6.89E-04	2.30E-04	0.01
KEGG_PATHWAY	hsa04914:Progesterone-mediated oocyte maturation	14	0.18	8.26E-05	HSP90AA1, CDC25C, CDC25A, CDC25B, CCNB1, CCNB2, MAD2L1, KRAS, ADCY9, PLK1, BUB1, MAD2L2, PIK3R3, CCNA2	225	86	5085	3.68	0.01	0	0.1
KEGG_PATHWAY	hsa04115:p53 signaling pathway	10	0.13	0	CCNE2, CCNB1, CCNE1, CDKN2A, CCNB2, RRM2, TSC2, CHEK1, PMAIP1, GTSE1	225	68	5085	3.32	0.32	0.07	3.16
KEGG_PATHWAY	hsa05222:Small cell lung cancer	11	0.14	0	E2F1, CCNE2, E2F2, CCNE1, CKS1B, E2F3, PTK2, SKP2, PIAS2, PIK3R3, ITGA2B	225	84	5085	2.96	0.4	0.08	4.12
KEGG_PATHWAY	hsa04360:Axon guidance	14	0.18	0	PLXNA1, EFNB3, PLXNA2, DPYSL5, EPHB1, PTK2, KRAS, UNC5B, PAK2, UNC5A, FYN, GSK3B, SRGAP1, SRGAP2	225	129	5085	2.45	0.45	0.08	4.83
KEGG_PATHWAY	hsa00240:Pyrimidine metabolism	11	0.14	0.01	PRIM1, TYMS, POLR3K, POLE2, RRM2, RRM1, DCK, POLA1, POLA2, NME7, TK1	225	95	5085	2.62	0.71	0.14	9.63
KEGG_PATHWAY	hsa05219:Bladder cancer	7	0.09	0.01	E2F1, RPS6KA5, E2F2, E2F3, CDKN2A, KRAS, PGF	225	42	5085	3.77	0.74	0.14	10.67
KEGG_PATHWAY	hsa05215:Prostate cancer	10	0.13	0.02	E2F1, CCNE2, E2F2, CCNE1, E2F3, HSP90AA1, KRAS, GSK3B, PIK3R3, CTNNB1	225	89	5085	2.54	0.9	0.2	17.14
KEGG_PATHWAY	hsa00230:Purine metabolism	14	0.18	0.02	POLR3K, POLA1, DCK, POLA2, HPRT1, GMPS, NME7, GART, PRIM1, ADCY9, POLE2, RRM2, PKLR, RRM1	225	153	5085	2.07	0.91	0.2	18.08
KEGG_PATHWAY	hsa03410:Base excision repair	6	0.08	0.02	POLE2, UNG, LIG1, MBD4, NTHL1, FEN1	225	35	5085	3.87	0.92	0.19	18.86
KEGG_PATHWAY	hsa05214:Glioma	8	0.1	0.02	E2F1, E2F2, E2F3, CDKN2A, KRAS, CALML3, CAMK2B, PIK3R3	225	63	5085	2.87	0.94	0.2	21.16
KEGG_PATHWAY	hsa05200:Pathways in cancer	23	0.3	0.03	E2F1, E2F2, FZD8, CKS1B, MSH6, E2F3, HSP90AA1, PGF, FGF9, SKP2, BIRC5, FZD2, CTNNB1, CTNNA2, CCNE2, CCNE1, PTK2, CDKN2A, KRAS, GSK3B, PIAS2, PIK3R3, ITGA2B	225	328	5085	1.58	0.99	0.27	30.48
KEGG_PATHWAY	hsa00670:One carbon pool by folate	4	0.05	0.03	TYMS, MTHFD2, SHMT2, GART	225	16	5085	5.65	0.99	0.26	31.03
KEGG_PATHWAY	hsa04916:Melanogenesis	9	0.12	0.07	FZD8, KRAS, ADCY9, CALML3, GSK3B, GNAS, CAMK2B, FZD2, CTNNB1	225	99	5085	2.05	1	0.47	57.19
KEGG_PATHWAY	hsa05210:Colorectal cancer	8	0.1	0.08	FZD8, MSH6, KRAS, GSK3B, BIRC5, FZD2, PIK3R3, CTNNB1	225	84	5085	2.15	1	0.48	60.57
KEGG_PATHWAY	hsa03430:Mismatch repair	4	0.05	0.08	RFC5, MSH6, RFC4, LIG1	225	23	5085	3.93	1	0.48	61.82
KEGG_PATHWAY	hsa05223:Non-small cell lung cancer	6	0.08	0.09	E2F1, E2F2, E2F3, CDKN2A, KRAS, PIK3R3	225	54	5085	2.51	1	0.5	66.23
KEGG_PATHWAY	hsa05218:Melanoma	7	0.09	0.09	E2F1, E2F2, E2F3, CDKN2A, KRAS, FGF9, PIK3R3	225	71	5085	2.23	1	0.5	67.79
KEGG_PATHWAY	hsa05212:Pancreatic cancer	7	0.09	0.1	E2F1, E2F2, E2F3, CDKN2A, KRAS, PGF, PIK3R3	225	72	5085	2.2	1	0.5	69.77

Genes comprising signature 3 (Additional file 7: Table S5) were uploaded into the DAVID gene ontology search engine (http://david.abcc.ncifcrf.gov/). KEGG pathway enrichment was generated and the table represents the output file ranked based on significance and annotated by column header.

**Table 4:** **KEGG pathway enrichment analysis for the genes comprising signature 3 (101.6.4)**

**Category**	**Term**	**Count**	**%**	**PValue**	**Genes**	**List Total**	**Pop Hits**	**Pop Total**	**Fold Enrichment**	**Bonferroni**	**Benjamini**	**FDR**
KEGG_PATHWAY	hsa00100:Steroid biosynthesis	3	0.1	0.03	SQLE, FDFT1, SC4MOL	86	17	5085	10.43	0.97	0.97	30.81
KEGG_PATHWAY	hsa05200:Pathways in cancer	11	0.38	0.05	LAMA1, HRAS, PTK2, SOS1, CBL, VEGFA, PPARG, RALA, LEF1, MDM2, LAMB1	86	328	5085	1.98	0.99	0.93	41.08
KEGG_PATHWAY	hsa04510:Focal adhesion	8	0.27	0.05	LAMA1, HRAS, PTK2, FLT1, DIAPH1, SOS1, VEGFA, LAMB1	86	201	5085	2.35	1	0.85	44.04
KEGG_PATHWAY	hsa00330:Arginine and proline metabolism	4	0.14	0.06	ARG1, P4HA2, P4HA1, CPS1	86	53	5085	4.46	1	0.82	49.31
KEGG_PATHWAY	hsa05216:Thyroid cancer	3	0.1	0.08	HRAS, PPARG, LEF1	86	29	5085	6.12	1	0.86	63

Genes comprising signature 4 (Additional file 8: Table S6) were uploaded into the DAVID gene ontology search engine (http://david.abcc.ncifcrf.gov/). KEGG pathway enrichment was generated and the table represents the output file ranked based on significance and annotated by column header.

Discrimination between cancer and benign control tissue and also between metastatic disease and other clinical cases represents an important goal of biomarker research. Thus, we used these gene signatures to classify clinical samples in prostate cancer samples and metastatic tissue samples in two additional datasets. One consisted of prostate cancer samples isolated by laser capture microdissection generated by Tomlins *et al.* [5] and the other contained expression array data from primary and metastatic tumours from multiple tissue sites generated by Ramaswamy *et al.* [6].

**Figure 1:**
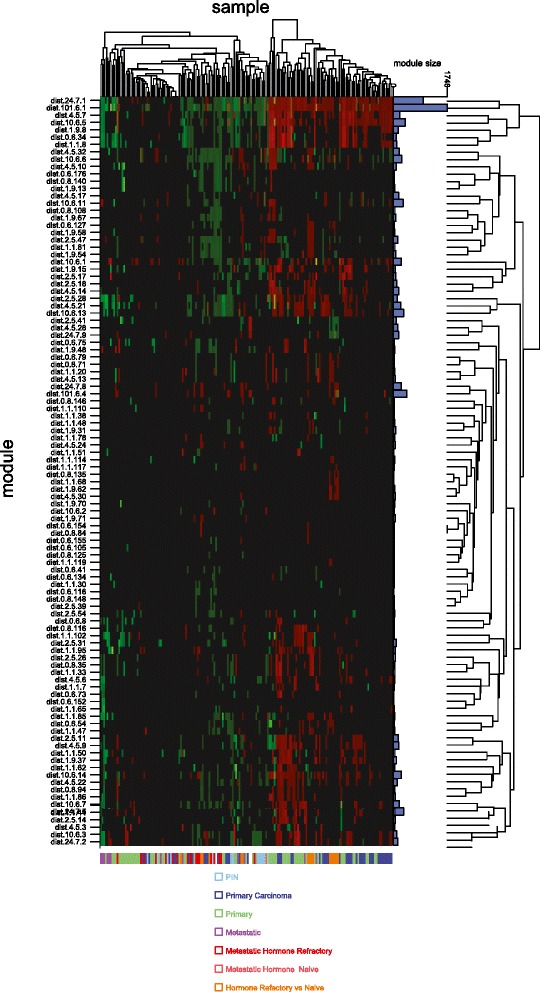
**Gene signatures capable of discriminating between prostate cancer subgroups and classify metastatic disease.** Gene signatures generated using the Varambally dataset and found to be significant discriminators of metastatic disease and primary/localised cancers (Additional file 10: Table S8) when applied to the Tomlins and Rawaswamy datasets were used to cluster samples in these datasets in a heatmap. The gene signatures represented are those capable of characterising samples from at least one progression stage (Fischer’s exact < = 0.05). Gene signatures are rows and samples are columns. The colour coded bar at the base of the heatmap indicates the clinical grouping for each sample as also defined in the key. Metastatic hormone refractory, metastatic hormone naïve and hormone refractory vs. naïve represent prostate cancer cases from the Tomlins dataset, as do PIN (prostatic intraepithelial neoplasia) and primary carcinoma. The other categories (metastatic and primary) are samples from the Rawaswamy dataset and are metastatic and primary cancers from multiple organ sites, not simply the prostate gland. The blue bar graph on the right-hand side of the heatmap depicts the number of genes in each signature which are differentially expressed and contribute to the sample clustering in this analysis. For signature 1 (dist 101.6.1 and Additional file 5: Table S3) this is 1748 genes in total as highlighted and other bars are numbers of genes relative to this. The colour scale represents the mean log2 fold change for differential gene signatures (> = abs log2(2)). Red indicates module induction, green repression. Gene signatures significant in both directions are indicated in yellow. Using the mean module log2 fold change we clustered the samples and modules using hierarchical clustering with euclidean distance as a measure of dissimilarity. Data points that contained both induced and repressed values have been excluded from the clustering.

The Tomlins dataset consisted of various refined subgroups based on isolation of cell sub-populations including stromal fractions, epithelial fractions, localised prostate cancer and hormone-naïve and refractory metastatic disease. The Ramaswamy *et al.* dataset consisted of cancers from 14 organ sites with paired normal samples as well as normal tissues. In each dataset, we asked whether our signatures and sub-signatures could discriminate between the sample groups. To determine this, we first assessed the mean fold-change in the expression of each gene signature in each sample group in both datasets (Additional file 9: Table S7). We then performed a Fischer’s Exact test to identify signatures that were capable of discriminating between localised prostate cancer, metastatic prostate cancer and the other sample groups defined in all three published studies – Varambally *et al.,* Tomlins e*t al.,* and Ramaswamy *et al.* (refer to Materials and Methods for more detail on subgroups/sample types) (Additional file 10: Table S8). Gene ontologies were assigned to these statistically significant gene clusters and the clustering is represented in a heatmap for the classifying modules combining both the Tomlins and Ramaswamy sample sets (Figure 1 and Additional files 10 and 11: Tables S8 and S9 for gene ontology annotations). The smallest gene signature (dist.0.6.34) capable of subclustering localised prostate cancer from other samples in all three datasets consisted of 71 genes (Additional file 12). This small signature was a sub-component of the original signature 1 (101.6.1). The most significantly enriched biological process associated with these genes was vascular smooth muscle contraction (hsa04270: Vascular smooth muscle contraction, p-value 2x10^−3^) (Table 5). The four genes within this signature that were individually most significantly overexpressed in localised prostate cancers compared to benign tissues and metastatic cases were an oncogenic transcription factor, v-myc avian myelocytomatosis viral oncogene homolog (MYC), a proteoglycan capable of sequestering transforming growth factor beta called fibromodulin (FMOD), a mitochondrial enzyme associated with fatty acid metabolism called glycine N-acyltransferase-like protein 1 (GLYATL1) and an extraneuronal monoamine transporter called solute carrier family 22 member 3 (SLC22A3). MYC has been shown to be overexpressed in prostate cancer [18] and to drive tumourigenesis in a transgenic model of the disease [19]. Fibromodulin has not been widely studied in cancer and has not been implicated in prostate cancer. It is, however, known to be significantly overexpressed in chronic lymphocytic leukemia (CLL) versus normal B lymphocytes [20] and associated with a resistance signature to DNA damage-induced apoptosis in CLL [21]. Furthermore, the expression of fibromodulin is known to be induced in leiomyoma in response to TGF-beta through Smad and MAP kinase signalling [22]. GLYATL1 has not been associated with cancers. SLC22A3 has been reported to be overexpressed in localised prostate cancer at the transcript level when compared to benign tissue [5].

**Table 5:** **KEGG pathway enrichment for the 71-gene signature capable of subclustering localised prostate cancer cases across multiple datasets**

**Category**	**Term**	**Count**	**%**	**PValue**	**Genes**	**List Total**	**Pop Hits**	**Pop Total**	**Fold Enrichment**	**Bonferroni**	**Benjamini**	**FDR**
KEGG_PATHWAY	hsa04270:Vascular smooth muscle contraction	5	6.94	0	ACTG2, MYH11, KCNMB1, MYLK, MYL9	26	112	5085	8.73	0.12	0.12	1.99
KEGG_PATHWAY	hsa05414:Dilated cardiomyopathy	4	5.56	0.01	DES, PLN, IGF1, TPM2	26	92	5085	8.5	0.47	0.27	9.6
KEGG_PATHWAY	hsa04960:Aldosterone-regulated sodium reabsorption	3	4.17	0.02	IGF1, ATP1A2, IRS1	26	41	5085	14.31	0.66	0.31	15.92
KEGG_PATHWAY	hsa04310:Wnt signaling pathway	4	5.56	0.04	SFRP1, CAMK2G, PRICKLE2, MYC	26	151	5085	5.18	0.91	0.45	31.53
KEGG_PATHWAY	hsa05410:Hypertrophic cardiomyopathy (HCM)	3	4.17	0.06	DES, IGF1, TPM2	26	85	5085	6.9	0.99	0.57	49.28

Genes were uploaded into the DAVID gene ontology search engine (http://david.abcc.ncifcrf.gov/). KEGG pathway enrichment was generated and the table represents the output file ranked based on significance and annotated by column header.

**Table 6:** **KEGG pathway enrichment analysis for the entire set of overexpressed genes in localised prostate cancer versus benign tissue in the Varambally dataset (GSE3325)**

**Category**	**Term**	**Count**	**%**	**PValue**	**Genes**	**List Total**	**Pop Hits**	**Pop Total**	**Fold Enrichment**	**Bonferroni**	**Benjamini**	**FDR**
KEGG_PATHWAY	hsa00512:O-Glycan biosynthesis	3	0.34	0.01	GALNTL4, GCNT1, ST6GALNAC1	26	30	5085	19.56	0.43	0.43	8.95
KEGG_PATHWAY	hsa04610:Complement and coagulation cascades	3	0.34	0.04	C4A, C4B, SERPINA1	26	69	5085	8.5	0.94	0.75	36.76
KEGG_PATHWAY	hsa05322:Systemic lupus erythematosus	3	0.34	0.08	C4A, C4B, HLA-DMB	26	99	5085	5.93	1	0.83	58.75

Genes were uploaded into the DAVID gene ontology search engine (http://david.abcc.ncifcrf.gov/). KEGG pathway enrichment was generated and the table represents the output file ranked based on significance and annotated by column header.

**Figure 2:**
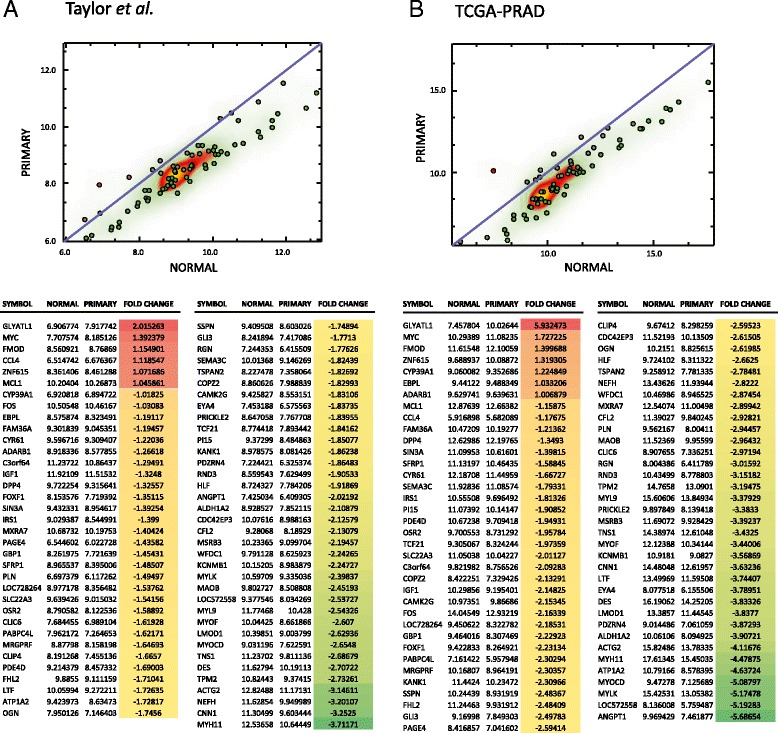
**Differential expression of a 71-gene signature classifier in a prostate cancer exon-array dataset (Taylor** ***et al.*** **) and the TCGA RNA-seq dataset for prostate cancer (TCGA-PRAD).** The expression values of the 71-gene signature (dist.0.6.34) capable of subclustering localised prostate cancer from other samples in all three interrogated datasets are shown in two independent datasets, **A**. a prostate cancer exon-array dataset (Taylor *et al.*) and **B**. TCGA RNA-seq dataset for prostate cancer (TCGA-PRAD) were used. Values were log2 normalized and the mean of the sample groups (PRIMARY TUMOUR/SOLID TISSUE NORMAL) is shown.

The other genes within this coexpression signature were downregulated in prostate cancers versus benign tissue and the majority were myosins, such as myosin, heavy polypeptide 11, smooth muscle (MYH11), myocardin (MYOCD), and myosin, light chain 9, regulatory (MYL9)thus accounting for the pathway enrichment for vascular smooth muscle contraction. As prostate cancer progresses to more advanced stages there is a depletion of stromal cells from the tissue and this perhaps explains the dominant contribution from downregulated muscle-associated genes to the signature and also other features of pathway enrichments particularly of the focal adhesion classification [23]. In order to determine whether our signature was consistent across more recent datasets, we downloaded an exon-array dataset generated by Taylor *et al.,* and also The Cancer Genome Atlas (TCGA) data recently generated using high-throughput transcript sequencing of prostate cancers [24] (data generated by the data generated by the TCGA Research Network: http://cancergenome.nih.gov/). MYC and GLYATL1 remain significantly overexpressed features (>1.3 fold) within these signatures in both datasets (Figure 2) with the vast majority of other gene transcripts downregulated including those enriched in the KEGG pathway analysis for vascular smooth muscle contraction.

Whilst our 71 gene signature mainly contains differentially expressed genes that are downregulated in cancers versus benign tissues, most prostate cancer biomarkers that are currently under evaluation are overexpressed transcripts and proteins in the disease state. Consequently, we next sought to evaluate genes that were overexpressed in localised prostate cancers in signatures 1-4 more thoroughly in other datasets. There were 97 annotated gene transcripts in total overexpressed (Additional file 13). We had previously performed pathway analyses on signatures 1-4 which included both up- and downregulated genes (Tables 1, 2, 3 and 4). We now repeated this solely for the 97 overexpressed genes and this yielded pathway enrichment for O-glycan biosynthesis (hsa00512: O-glycan biosynthesis, p-value 0.009) as the most significant KEGG enrichment (Table 6).

**Table 7:** **Comparison of the performance of a 31-gene signature with ERG, AR and KLK3 in discriminating between benign tissue, localised prostate cancer and metastatic disease**

**Gene/signature**	**AUC benign-local**	**AUC benign-metastatic**	**AUC localized-metastatic**
KLK3	0.5204082	0.9104938	0.8707483
ERG	0.812616	0.9326599	0.6099773
AR	0.6581633	0.8395062	0.8435374
31 Gene signature	0.994898	0.9938272	0.957672
Derived from the Grasso Data			

Data were downloaded from Grasso *et al.,*. ROC statistics were computed in an evaluation sample set having established the weighting for genes in the signature using logistic regression in a test sample set. We report the area under the curve (AUC) for each transcript and for the signature for each of three pairwise comparisons as generated using the R package ROCR.

To further refine this gene set, we then interrogated the Oncomine compendium of expression array data to determine which of these 97 genes are significantly overexpressed in at least three additional independent prostate cancer datasets when a Top 1% overexpression threshold was applied together with a p-value threshold of 1x10^−4^ [25]. Thirty three annotated genes, around one-third of the 97-gene set fulfilled these criteria. This included 3 of the 4 overexpressed genes (MYC, GLYATL1 and SLC22A3) in the 71 gene signature subgrouping prostate cancers in the Varambally, Tomlins and Ramaswamy studies (highlighted in bold in Additional file 13). This 33 gene set also included four of the five glycosylating enzymes (UDP N-acetylglucosamine pyrophosphorylase 1 (UAP1), glucosaminyl (N-acetyl) transferase 1, core 2 (GCNT1), beta-1,3-glucuronyltransferase 1 (B3GAT1) and RAP1 GTPase activating protein 2 (RAP1GAP2/GARNL4)) contributing to the ontology enrichment for glycan biosynthesis in the larger 97 gene set. Notably, others and we have recently reported that UAP1 and GCNT1 are overexpressed in prostate cancer tissue using immunohistochemistry. In addition, an aminosugar conjugate, O-linked N-acetylglucosamine (O-GlcNAc), is also significantly elevated in prostate cancer [26,27]. Furthermore, the UAP1 transcript has also been reported to be detectable in urine and plasma samples as a component of a multi-gene signature [28]. Additionally, UDP sugar conjugates have been identified as elevated in prostate cancers through metabolomics and O-linked N-acetylglucosamine is an overexpressed prostate cancer tissue biomarker, which can be conjugated to a variety of proteins to affect their stability and activity including c-Myc [29,30]. Consequently, the presence of these genes encoding glycosylating enzymes within this signature has been partly validated in tissue at the proteins level and suggests that more systematic profiling of glycoproteins may reveal new biomarkers.

Biologically, it is interesting to consider what might contribute to the increased expression of these genes in prostate cancers. Prostate cancer is driven by the dysregulated expression and activity of a number of transcription factors. The most notable example is the androgen receptor but others are overexpressed through chromosomal rearrangements and gene fusions as well as copy number variation as prostate cancer develops and progresses. This in turn has a significant impact on the expression of gene targets for these transcription factors and makes it plausible that a proportion of overexpressed genes reflect changes in transcription factor expression and activity. In this context, it is noteworthy that a total of five transcription factors were present in this group of 33 annotated genes ((single-minded family bHLH transcription factor 2(SIM2), MYC, distal-less homeobox 1 (DLX1), homeobox C6 (HOXC6) and v-ets avian erythroblastosis virus E26 oncogene homolog (ERG)).

c-Myc is a well-established oncogenic transcription factor, which is overexpressed through chromosomal amplification on 8q24 but also through post-translational events, which may include glycosylation of the N-terminal transactivation and concomitant antagonism of proteasomal degradation [18,31,32]. ERG is part of a highly prevalent gene fusion affecting chromosome 21 and driven by the activity of the AR [33]. It is overexpressed in around 50% of prostate cancers through a chromosomal rearrangement, which fuses it to the upstream androgen receptor-dependent regulatory element controlling TMPRSS2 expression. SIM2 overexpression is associated with changes in transcriptional control affecting other loci on chromosome 21 [34,35]. Target genes for MYC and ERG have been extensively explored in clinical and cell-line datasets using expression array profiling with targeted knockdown and overexpression in prostate cancer cells. These approaches have linked MYC to processes including ribosome biogenesis and splicing and ERG to cell motility and migration, respectively [36-38]. Whilst the 33 genes did not include significant number of established MYC target genes, several reported ERG target were present including B3GAT1, phospholipase A1 member A (PLA1A) and collagen, type IX, alpha 2 (COL9A2) [39]. In addition, there were a number of direct AR targets including UAP1 and GCNT [30,40]. Importantly, whilst the AR is the principal transcription factor driving all stages of prostate cancer development, its target genes cannot be easily inferred by coexpression with the AR in contrast to ERG relative to ERG target genes. Target genes for HOXC6, SIM2 and DLX1 are less well defined in prostate cancers but given the presence of ERG and AR target genes within this geneset it is highly likely that they also contribute, being transcription factors, to the expression of some of these genes. A more systematic understanding of the interplay between these transcription factors and dependent gene networks will emerge in future studies. This will require targeting the expression of the transcription factors in experimental model systems and profiling concomitant changes in transcription factor recruitment, chromatin architecture and gene expression.

**Figure 3:**
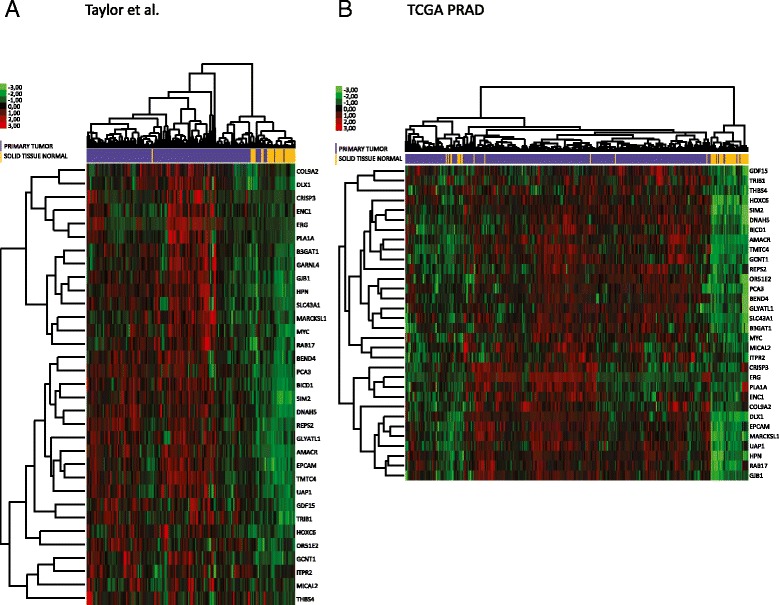
**Heatmaps confirming the clustering ability of the 33-gene signature in a prostate cancer exon-array dataset (Taylor** ***et al.*** **) and the TCGA RNA-seq dataset for prostate cancer (TCGA-PRAD).** The 33-gene signature was applied to two independent datasets, **A**. a prostate cancer exon-array dataset (Taylor *et al.*), and **B**. TCGA RNA-seq dataset for prostate cancer (TCGA-PRAD). Expression values were log2 transformed, normalized for high level mean and variance and hierarchically clustered using Euclidian distance. Genes are rows and samples are columns. The colour coded bars indicate expression values and the clinical grouping for each sample as defined in the keys.

In the interim, however, it was possible to infer co-dependency based on co-clustering of genes in clinical samples. We did so in two additional datasets, an exon-array dataset generated by Taylor *et al.* and a transcriptomic dataset generated for prostate cancer through high-throughput sequencing by the The Cancer Genome Atlas (TCGA) (Figure 3). In both datasets, we were able to firstly reconfirm the ability of these 33 genes to discriminate between localised prostate cancer and benign tissue samples (Figure 3). Secondly, ERG co-clustered within these 33 genes with bona fide target genes such as B3GAT1 and PLA1A corroborating a contribution at least from ERG to this prostate cancer-specific overexpression signature [39]. Intriguingly, another transcription factor, DLX1, also co-clustered with ERG raising the possibility of a transcription factor hierarchy in which early emergence of an ERG gene fusion may trigger aberrant expression of other developmental transcription factors.

Currently prostate-specific antigen (PSA)/kallikrein 3 (KLK3) is the most widely used protein biomarker for prostate cancer. The androgen receptor (AR) is the most significant transcription factor driving prostate cancer, but is also expressed at high levels in untransformed luminal epithelial cells and therefore is predominantly used as a transcript biomarker associated with metastatic disease and concomitant copy-number amplification [41]. Gene fusions have been detected which significantly elevate the transcript levels of ETS transcription factors and the most prevalent example in prostate cancer is the TMPRSS2-ERG fusion [33]. Detection of the fusion has been reported in biological fluids including urine samples [42].

**Figure 4:**
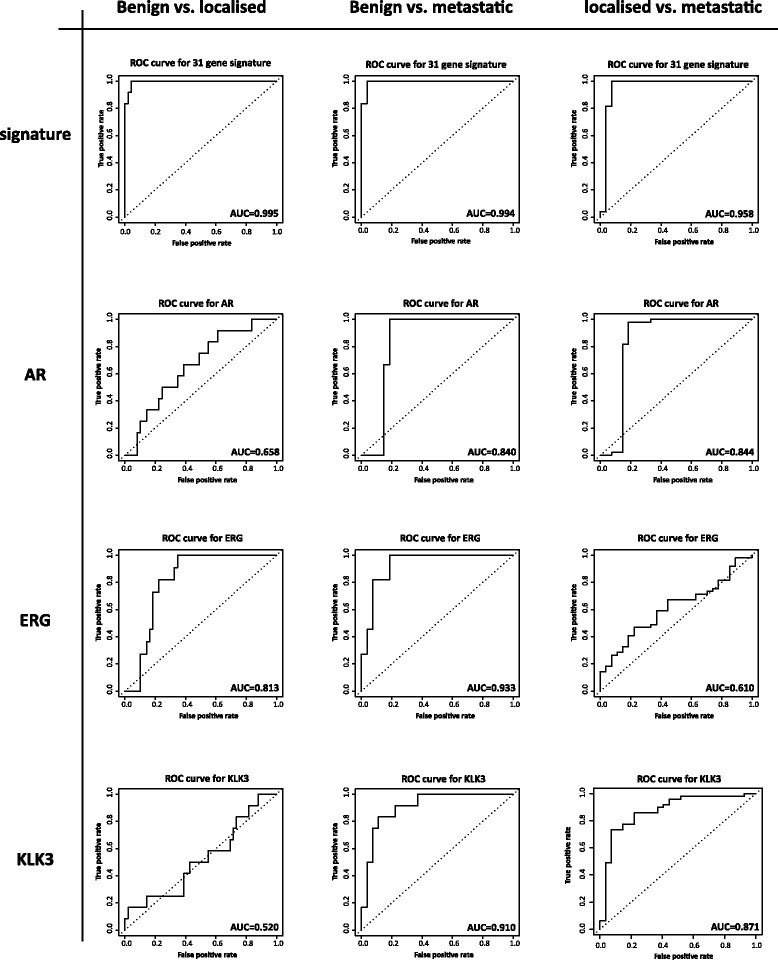
**Receiver operating characteristic (ROC) curves for discrimination between localised prostate cancer and benign cases, metastatic and benign cases and metastatic and prostate cancers using a 31-gene signature (row 1), AR (row 2), ERG (row 3) and KLK3 (row 4).**

To assess the performance of the 33-gene signature in comparison to KLK3/PSA, AR and ERG we interrogated an additional independent expression array dataset generated by Grasso *et al.,* and consisting of benign tissue, localised prostate cancer and metastatic cases [43]. This dataset was generated using two different array platforms on distinct sets of samples (Methods section). Cysteine-rich secretory protein-3 (CRISP3) was excluded from the signature due to missing values in the datasets for this gene and prostate cancer antigen 3 (PCA3) was not represented on the arrays leaving a 31-gene signature for evaluation. In the first phase of the signature evaluation we assessed the weighted contribution of each gene in the signature using a logistical regression model on a training dataset consisting of the samples profiled on an Agilent oligo microarray platform. We then used the samples profiled on a second platform, the 4x44K Agilent microarray to evaluate the performance of the signature and compared this to KLK3, ERG and AR. Three pairwise sample comparisons were undertaken - benign versus localised prostate cancer, benign versus metastatic cases and localised prostate cancers versus metastatic cases. Whilst all three transcripts and the signature discriminated between metastatic samples and benign tissue with good specificity and sensitivity as reflected in an area-under-the-curve (AUC) ranging from 0.83 for the AR to 0.99 for the signature, only the signature provided an AUC of ≥ 0.95 for all three pairwise comparisons (Table 7). Since both the AR and KLK3 are expressed in both untransformed prostate cells and prostate cancer it is perhaps not surprising that neither yielded an AUC of >0.65 in discriminating between localised prostate cancer and benign tissue samples. By contrast ERG expression is driven by a cancer-associated gene fusion and the AUC was 0.81 (Table 7). AR is amplified and overexpressed in metastatic prostate cancers and this likely explains the higher AUC for this marker (0.84) in discriminating metastatic cases from localised prostate cancers [41]. KLK3/PSA was also higher, 0.87, in this context. ERG by contrast whilst consistently overexpressed in the majority of localised prostate cancers is of variable utility as a prognostic marker according to the study cohort examined associating variously positively or negatively with progression and metastasis [44-46]. In our evaluation the AUC for ERG in discriminating localised prostate cancer from metastatic cases was 0.61, performing more poorly than as a discriminator of localised prostate cancer from benign tissue samples. The AUC differences between the markers and the signature in each pairwise comparison of the sample sets was also visualised in receiver operating characteristic (ROC) curves (Figure 4). These comparisons highlight the importance of using a multi-gene signature since no single gene provides robust discrimination at all stages of the disease, no doubt reflecting changes in the underlying biological drivers during disease progression. We provide in addition AUC values for each individual gene and array probe for each of the pairwise sample comparisons in the test set (Additional file 14: Table S12) and the validation set (Additional file 15: Table S13). Although beyond the scope of this paper we hope that this will assist in further evaluation of the signature by researchers in the field.

## Conclusions

**Figure 5:**
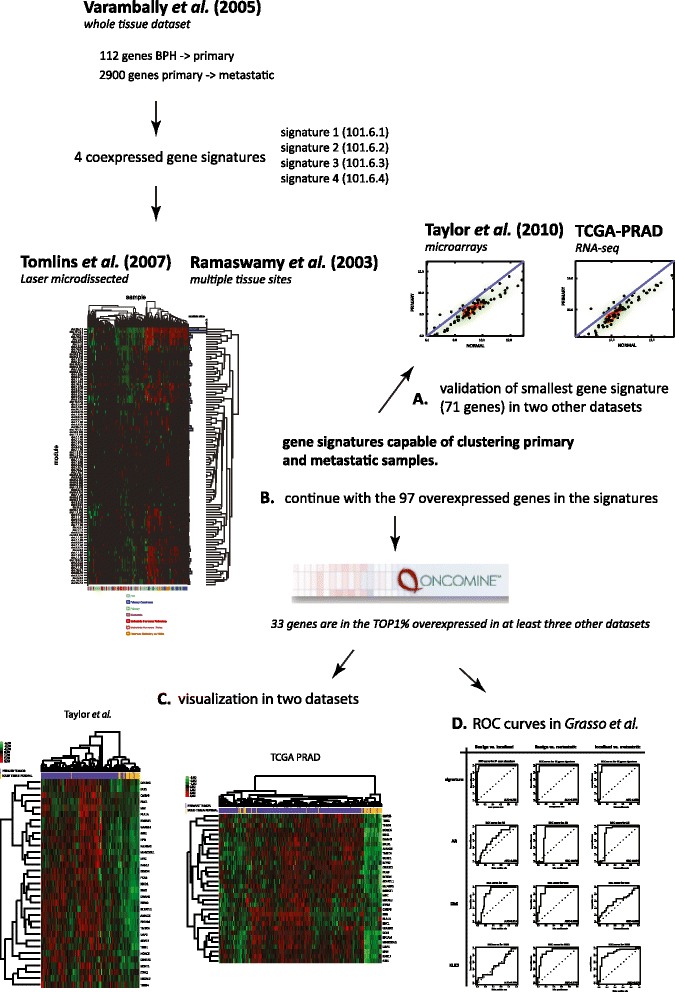
**Workflow for the identification of robust gene signatures and gene sets for clustering prostate cancer cases.** In step 1, we identified all statistically significant differentially expressed Affymetrix array probes in a small dataset consisting of 13 macrodissected clinical samples encompassing localised benign prostatic hyperplasia, localised prostate cancer and metastatic disease (GSE3325). We then generated gene signatures from these based on gene coexpression at varying stringency thresholds. These gene signatures were then applied to two additional datasets, a microdissected dataset (Tomlins et *al.*) and a multi-tissue site cancer and metastatic dataset (Ramaswamy *et al.*). A large number of the coexpression gene signatures clustered localised prostate cancers from metastatic disease and prostate metastases from other sample sets. The most compact gene signature able to do so consisted of 71 genes **(A)** and we assessed its expression pattern in two additional datasets, an exon-array dataset (Taylor *et al.*) and in a RNA-sequenced dataset (TCGA-PRAD). Few of the genes in the significant coexpression gene signatures were overexpressed genes in localised prostate cancers. In the second phase of the study, we abstracted all of the overexpressed genes and refined this down to a set of 33 genes based on significant overexpression in additional publicly available prostate cancer microarray datasets housed within the Oncomine database **(B)**. These genes also effectively clustered benign versus cancer cases in an exon-array dataset (Taylor *et al.*) an expression microarray dataset (Grasso *et al.*) and a RNA-sequenced dataset (TCGA-PRAD) **(C and D)**. In conclusion, it is possible to generate gene classifiers of clinical prostate cancer from a small dataset of macrodissected samples with the capacity to classify larger sequenced and microdissected datasets based on clinical characteristics.

In conclusion, in this study we have used a multi-step approach to refine gene signatures derived from diverse transcript detection platforms and sample types in order to arrive at a robust gene signature able to discriminate between PCa and benign tissue (Figure 5). This is the first time that this has been attempted and demonstrates that value exists in transcript signatures generated from amongst the earliest microarray studies right through to high-throughput sequencing. In brief, beginning with a small expression array dataset consisting of 13 macrodissected samples, we have been able to derive gene signatures capable of subclustering localised PCa and metastases in a larger microdissected and a multi-cancer dataset (Figure 5). This highlights that there are valuable gene transcript signatures that can be robust despite cellular heterogeneity in PCa and the evolution of transcript detection technologies. In addition, we have discovered that gene transcripts that are significantly overexpressed within these signatures are also overexpressed in much more recently acquired exon-array and sequence-based TCGA data, transcript detection platforms that were unavailable when the Varambally, Tomlins and Ramaswamy studies were undertaken (Figure 5). Finally, we have evaluated the performance of these transcripts as a signature in discriminating between benign tissue samples, localised PCa and metastatic disease in an additional dataset generated by Grasso *et al.* ROC curves reveal that the signature exceeds the performance of ERG, KLK3 or the AR as a classifier. Intriguingly, one third of these genes are glycosylating enzymes and transcription factors. PCa is significantly driven by a transcription factor, the AR, but there is increasing evidence of contributions by others and of interplays between them and indeed our signature does not include the AR itself. However, it includes both established examples (MYC and ERG) but also others that have so far been less studied (SIM2, DLX1 and HOXC6). Mechanistically, future work will investigate this transcriptional co-dependency in more detail and clinically these signatures will be further evaluated in clinical cohorts.

## Additional files

Additional file 1: Figure S1.
*W(C)* values generated from branch length thresholds plotted against the number of clusters produced for single, average, complete, ward and mcquitty agglomerative hierarchical and divisive clustering methods. Additional file 2: Table S1. Annotated Affymterix probes for differentially expressed genes in localised prostate cancer and metastatic tumours. Genes differentially expressed within dataset GSE3325. Columns 1 and 2 represent Affymetrix probe identifiers and gene symbols, respectively. Columns 3-5 represent mean probe signals for benign, prostate cancer and metastatic samples. Columns 6-9 represent a linear fold change and corresponding p-value for each contrast. Genes are ranked based on overexpression in localised prostate cancer versus benign tissue. Differential expression and associated statistical significance (p-values) have been calculated as described in the methods/supplementary methods section. Additional file 3: Figure S2.
*H(X)* GO bit values generated from branch length thresholds plotted against the number of clusters produced for single, average, complete, Ward and Mcquitty agglomerative hierarchical and divisive clustering methods. Additional file 4: Table S2. Differentially expressed genes within Varambally dataset (accession number GSE3325) grouped into coexpression signatures using the Ward agglomerative method. Genes are ranked based on overexpression in localised prostate cancer versus benign tissue. Annotation includes the cytogenetic locus for each gene and the gene name. Coexpression modules are defined at multiple branch thresholds. Nine branch thresholds were used. Dist.0.6 represents the most compact modules at the highest stringency threshold and dist.101.6 representing the least compact modules at the lowest stringency threshold. Numbers attributed to each gene within each branch threshold define membership of a distinct coexpression module. With reference to the main text ‘signature 1’ corresponds to 101.6.1, ‘signature 2’ corresponds to 101.6.2, ‘signature 3’ corresponds to 101.6.3 and ‘signature 4’ corresponds to 101.6.4. Additional file 5: Table S3. Differentially expressed genes comprising signature 1. Genes within signature 1 and sub-signatures defined at more stringent branch thresholds are ranked based on overexpression in localised prostate cancer within dataset GSE3325. Annotation includes the cytogenetic loci, gene names and unigene modules. Additional file 6: Table S4. Differentially expressed genes comprising signature 2. Genes within signature 2 and sub-signatures defined at more stringent branch thresholds are ranked based on overexpression in localised prostate cancer within dataset GSE3325. Annotation includes the cytogenetic loci, gene names and unigene modules. Additional file 7: Table S5. Differentially expressed genes comprising signature 3. Genes within signature 3 and sub-signatures defined at more stringent branch thresholds are ranked based on overexpression in localised prostate cancer within dataset GSE3325. Annotation includes the cytogenetic loci, gene names and unigene modules. Additional file 8: Table S6. Differentially expressed genes comprising signature 4. Genes within signature 4 and sub-signatures defined at more stringent branch thresholds are ranked based on overexpression in localised prostate cancer within dataset GSE3325. Annotation includes the cytogenetic loci, gene names and unigene modules. Additional file 9: Table S7. Fold-change in coexpressed gene signatures derived from Varambally *et al.,* in samples from a laser capture microdissected prostate cancer dataset and a multi-cancer dataset. All signatures and sub-signatures generated from Varambally *et al.* were tested for enrichment in individual samples from two additional datasets – a laser-capture microdissected prostate cancer dataset generated by Tomlins *et al.,* and a multi-cancer dataset generated by Ramaswamy *et al.,* Column A and B are collectively signature identifiers indicating the threshold for coexpression (column A) and the signature/module number (column B). Column C indicates the overall direction of the fold-change indicating overexpression (‘induced’) or downregulation (‘repressed’). Only signatures achieving a fold-change of at least two-fold are listed. Columns C through to CX represent individual samples from the Tomlins study. The data are listed sample-by-sample in order of clinical progression beginning with prostatic intraepithelial neoplasia (‘PIN’) cases and progressing to localised prostate cancer (‘Primary Carcinoma’), followed by metastatic hormone naïve cases and finally metastatic hormone refractory samples. The GSM accession code provided for each sample is traceable through the NCBI GEO data repository from which the data were downloaded. Finally there are columns representing pairwise sample comparisons between metastatic hormone refractory and naïve samples. Fold-change information for the data for samples from Ramaswamy *et al.* is represented in column CY onwards. Additional file 10: Table S8. Significance values and overall direction of the differential expression of gene signatures derived from Varambally *et al.,* in samples from a laser capture microdissected prostate cancer dataset and a multi-cancer dataset. All gene signatures are classified with aggregate p-values, overall gene ontology assignments and a descriptor of overexpression (‘induced’) or underexpression (‘repressed’) normalised to benign specimens from GSE3325. Laser capture microdissected material is subgrouped as described in the original publication and in the methods section incorporating prostatic intraepithelial neoplasia (PIN), localised prostate cancer (PCA), hormone refractory (HR), metastatic hormone refractory (MET_HR) and metastatic hormone naïve disease (MET_HN). In addition gene signatures are defined as sub-signatures of larger signatures generated using lower stringencies/broader branching thresholds, the relationship between gene signatures at different thresholds is indicated in columns X-AE. The number of genes within each coexpression module is given in the column entitled ‘moduleSize.varambally’. The numbers of corresponding mapped genes in the other data sets are indicated in columns Y and W. Gene ontology assignments for each signature are provided in column AF. Additional file 11: Table S9. Gene composition and ontology assignments to statistically significant gene signatures. Sheet one lists the gene signatures and the genes that comprise them which are capable of clustering sample groups within the Tomlins and Ramaswamy datasets (Fischer’s exact test < 0.05 p-value). Sheet two lists the GO terms associated with each significant discriminatory gene signature (<0.05 p-value). The discriminatory groups shown here mirror those depicted in the heatmap in Figure 1. Sheet three summarises the GO terms that are significant discriminators of important clinical sample groupings within the Tomlins and Ramaswamy datasets. Additional file 12: Table S10. A 71-gene signature capable of subclustering localised prostate cancer cases across multiple datasets. Additional file 13: Table S11. Overexpressed genes in localised prostate cancer versus benign tissue within the entire set of differentially expressed genes in the Varambally dataset (GSE3325). Additional file 14: Table S12. AUC values for individual probes for ERG, KLK3, AR and the gene signature in the test dataset from Grasso et al. Additional file 15: Table S13. AUC values for individual probes for ERG, KLK3, AR and the gene signature in the evaluation dataset from Grasso et al.
